# Image quality and diagnostic accuracy of reduced-dose computed tomography enterography with model-based iterative reconstruction in pediatric Crohn’s disease patients

**DOI:** 10.1038/s41598-022-06246-z

**Published:** 2022-02-09

**Authors:** Yeoun Joo Lee, Jae-Yeon Hwang, Hwaseong Ryu, Tae Un Kim, Yong-Woo Kim, Jae Hong Park, Ki Seok Choo, Kyung Jin Nam, Jieun Roh

**Affiliations:** 1 Department of Pediatrics, Pusan National University Children’s Hospital, College of Medicine, Pusan National University, Yangsan, 50612 Republic of Korea; 2 Department of Radiology, Research Institute for Convergence of Biomedical Science and Technology, Pusan National University Yangsan Hospital, College of Medicine, Pusan National University, Yangsan, 50612 Republic of Korea

**Keywords:** Computed tomography, Crohn's disease, Paediatric research

## Abstract

This study assessed the image quality and diagnostic accuracy in determining disease activity of the terminal ileum of the reduced-dose computed tomography enterography using model-based iterative reconstruction in pediatric patients with Crohn’s disease (CD). Eighteen patients were prospectively enrolled and allocated to the standard-dose (SD) and reduced-dose (RD) computed tomography enterography (CTE) groups (*n* = 9 per group). Image quality, reader confidence in interpreting bowel findings, accuracy in determining active CD in the terminal ileum, and radiation dose were evaluated. Objective image quality did not show intergroup differences, except for image sharpness. Although reader confidence in detecting mural stratification, ulcer, and perienteric fat stranding of the RD-CTE were inferior to SD-CTE, RD-CTE correctly diagnosed active disease in all patients. The mean values of radiation dose metrics (SD-CTE vs. RD-CTE) were 4.3 versus 0.74 mGy, 6.1 versus 1.1 mGy, 211.9 versus 34.5 mGy∙cm, and 4.4 versus 0.7 mSv mGy∙cm for CTDI_vol_, size-specific dose estimation, dose-length product, and effective dose, respectively. RD-CTE showed comparable diagnostic accuracy to SD-CTE in determining active disease of the terminal ileum in pediatric CD patients. However, image quality and reader confidence in detecting ulcer and perienteric fat stranding was compromised.

## Introduction

Inflammatory bowel disease is characterized by chronic and debilitating inflammatory episodes of the gastrointestinal tract. Due to the increasing incidence of inflammatory bowel disease in the pediatric population^1^, imaging studies visualizing the bowel, including endoscopy, computed tomography enterography (CTE), and magnetic resonance enterography (MRE), have also been frequently performed in pediatric patients^2^. Computed tomography (CT) is an excellent noninvasive tool for evaluating abdominal manifestations. Moreover, patients diagnosed with Crohn’s disease (CD) in childhood are twice as likely to have high cumulative radiation exposure compared to patients diagnosed at an older age even though reduced-dose (RD) CT protocol is currently being used for pediatric patients in many institutions^3^.

A few technical parameters can be applied to minimize the radiation dose during a CT scan, including tube current modulation, peak kilovoltage (kVp) lowering, and the use of iterative reconstruction. Low kVp can increase contrast visualization, particularly in CT angiography, due to higher attenuation of iodine contrast media with lower tube voltage as photon energy decreases toward the K-edge energy of 33 keV^4^ while achieving radiation dose reduction^4–6^. However, low kVp CT images inherently increase quantum mottles because of the higher absorption of low-energy photons within the human body, particularly in larger patients^4^. Therefore, the low kVp CT scan may be suitable for pediatric patients because image noise and streak artifacts are reduced in small individuals^7^.

Model-based iterative reconstruction (MBIR; Veo, GE Healthcare, Milwaukee, WI, USA) enables drastic image noise reduction. MBIR is known to achieve a higher level of noise reduction of up to 60–80% of the standard filtered backprojection algorithm in adult patients with effective denoizing performance^8,9^. However, the main drawback of MBIR is associated with undesirable image features expressed as *blotchy*, *pixelated*, or *plastic-like* image texture compared with conventional filtered backprojection images.

Several studies have evaluated the feasibility and diagnostic accuracy of reduced-dose CTE (RD-CTE) in adult and pediatric patients by applying low kVp and iterative reconstruction^10–16^. These studies achieved a significant dose reduction of approximately 30%–70% with acceptable image quality and diagnostic accuracy in detecting bowel abnormalities. However, it is believed that few prospective studies have evaluated the accuracy of RD-CTE in assessing small bowel abnormalities in pediatric CD patients.

Therefore, the purpose of the current study was to assess the feasibility, image quality, and diagnostic accuracy in determining disease activity of the terminal ileum of the RD-CTE using low kVp and MBIR techniques in pediatric CD patients.

## Methods

### Patient enrolment

This prospective study was approved by the Institutional Review Board of Pusan National University Yangsan Hospital, Yangsan, Republic of Korea (IRB no: 04–2014-024). All methods were performed following the Declaration of Helsinki and HIPAA regulations. Written informed consent for CTE and ileocolonoscopy was obtained from each patient and their guardians. Inclusion criteria were as follows: patients between 9 and 18 years old, patients with clinically suspected or known CD, and patients clinically indicated to CTE and ileocolonoscopy to evaluate small bowel CD. The exclusion criteria were as follows: patients who had a contraindication for intravenous injection of iodinated contrast media or ileocolonoscopy, failure to evaluate the terminal ileum on ileocolonoscopy, patients who were intolerant to negative oral contrast media, patients who could not perform a 10-s breath-hold, and patients with high suspicion of bowel obstruction. Patients were grouped into the standard- or RD groups by a simple randomization method using computer-generated random numbers. Patients’ characteristics, clinical and laboratory findings (i.e., including pediatric CD activity index, C-reactive protein, and fecal calprotectin) were obtained by reviewing electrical medical records.

### CTE protocols and image reconstruction

Patients were required to fast for 6 h before the CTE. Negative oral contrast media (0.1% *w*/*v* barium solution; Easymark, TAEJOON PHARM Co. Ltd., Seoul, Korea) was administered 1 h before the CT scan. The total amount of negative oral contrast media were 1,000, 1,200, and 1,500 mL for patients weighing < 40, 40–59.9, and > 60 kg, respectively. In addition, 30%, 30%, 20%, and 20% of the total amount of negative oral contrast media was administered 60, 45, 30, and 15 min before the start of the CT examination, respectively. Moreover, no spasmolytic agents were used.

Administered intravenously through a 22-gage peripheral venous access in the antecubital vein was 1.5 cm^3^/kg of iopromide (Ultravist 370; Bayer Healthcare AG, Leverkusen, Germany). The upper limit of the total amount of contrast medium used was 100 mL. The contrast media injection rate was adjusted so that the contrast injection was completed in 35 s, followed by a saline chaser. Single-phase CT scanning was performed 50 s after contrast injection, and the scan was performed in the craniocaudal direction, starting from the dome of the liver and inferior margin of the symphysis pubis. CT scans were performed with the patient in the prone position using a 64-channel multidetector-row CT scanner (Discovery 750 HD, GE Healthcare). CT parameters were collimation (0.625 mm × 64), pitch (0.984:1), rotation time (0.5 s), matrix (512 × 512), and field-of-view, optimized for each patient. Moreover, automatic exposure control was applied.

Peak kilovoltage, noise index, and milli-ampere range were set according to the patients' weights (Supplementary Material 1). In addition, 80 kVp was applied for the RD-CTE regardless of body weight to reduce radiation dose and increase tissue contrast^4–6^. The noise index of the RD-CTE was empirically set based on the clinical experience of MBIR denoizing capability, the image quality of the upper abdomen of low-dose chest CT scan and low-dose abdominopelvic CT scans, and inverse proportional relationship between the noise index and the square root of the radiation dose^17,18^. In the SD-CTE group, eight and one CT scans were performed at 100 and 120 kVp, respectively.

**Figure 1:**
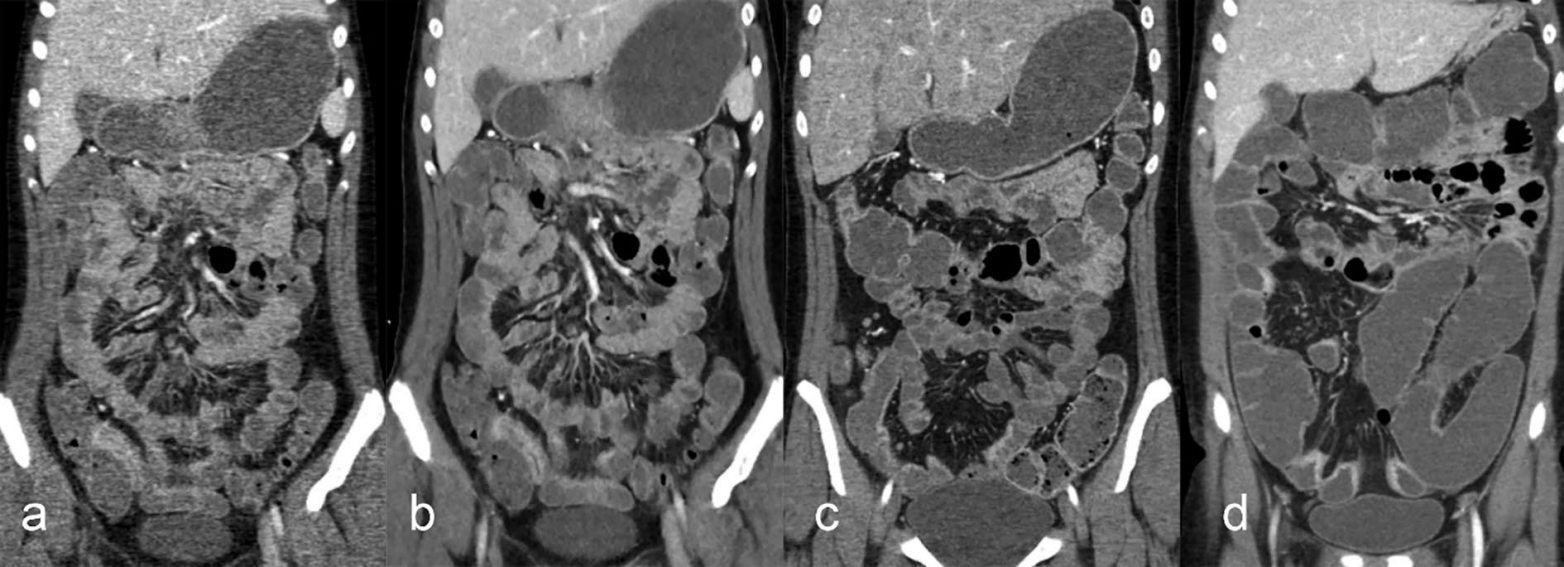
Representative images of CT enterography. (**a**) reduced-dose group with adaptive statistical iterative reconstruction with a blending factor of 100%, (**b**) reduced-dose group with model-based iterative reconstruction, and (**c** and **d**) standard-dose group (**a**, **b**, **c**, and **d**) represent 1 (inadequate for diagnosis), 2 (worse than routine examination, but interpretable), 3 (similar to routine examination), and 4 (better than routine examination), respectively. Note that images for (**a** and **b**) are the same patient.

Raw projection data were multiplanar reconstructed (axial and coronal planes) with adaptive statistical iterative reconstruction with a 50% blending factor for the SD-CTE and MBIR for the RD-CTE. The reconstruction thickness was 2.5 mm at a 2.5-mm slice interval, regarding the purpose of the CTE and radiation dose. Representative images for each group are shown in Fig. 1. The patients were discharged 30 min after the procedure and monitored for any related adverse effects.

### Image quality analysis

CT examinations were randomly reordered before image quality analysis and stored in the Picture Archiving and Communication System (Infinitt PACS M6, Infinitt Healthcare, Seoul, Korea) of the facility of the current study after removing patients' identifiable information. Standard abdominal (window width, 400 Hounsfield unit (HU); window level, 20 HU) window settings were used for image evaluation; however, reviewers were allowed to adjust window settings and magnification as per their personal visual preference.

### Objective image quality analysis

For the objective image quality analysis, one radiologist with 13 years of experience as a board-certified gastrointestinal radiologist selected two axial CT images at the level of and the ileocecal valve. Image noise, signal-to-noise ratio (SNR) and contrast-to-noise ratio (CNR) were obtained by placing a 15-mm circular region of interest on the right psoas muscle in the two axial CT images. Image noise was defined as the standard deviation of the Hounsfield units in the region of interest. The SNR and CNR values were calculated using the following formula:

$${\text{SNR}} = {\raise0.7ex\hbox{${{\text{Target}}\;{\text{HU}}}$} \!\mathord{\left/ {\vphantom {{{\text{Target}}\;{\text{HU}}} {{\text{Target}}\;{\text{SD}}}}}\right.\kern-\nulldelimiterspace} \!\lower0.7ex\hbox{${{\text{Target}}\;{\text{SD}}}$}}$$

$${\text{CNR}} = {\raise0.7ex\hbox{${{\text{Target}}\;{\text{HU}} - {\text{Background}}\;{\text{HU}}}$} \!\mathord{\left/ {\vphantom {{{\text{Target}}\;{\text{HU}} - {\text{Background}}\;{\text{HU}}} {{\text{Target}}\;{\text{SD}}}}}\right.\kern-\nulldelimiterspace} \!\lower0.7ex\hbox{${{\text{Target}}\;{\text{SD}}}$}}$$

where HU denotes the Hounsfield unit and SD denotes the standard deviation of the HU.

Objective measurement of the image sharpness was assessed using the *blur metric* analysis (MATLAB 2019a, Mathworks, Inc., Natick, MA, USA)^19^. The blur metric quantified image sharpness by comparing intensity variations between adjacent pixels of the original and low-pass-filtered images, and the calculated values were expressed as numeric values ranging from 0 to 1. Lower values indicate sharp images, and higher values represent blurred images^20,21^. Image sharpness was measured at two contiguous axial images at the ileocecal valve level, and the average values from the two images were regarded as representative values.

### Subjective image quality analysis

Subjective image quality was independently assessed by a radiologist with 10 years of experience as a board-certified pediatric radiologist and a radiologist with 4 years of experience as a board-certified gastrointestinal radiologist. The entire set of axial and coronal images was displayed on diagnostic quality PACS workstation monitors in random order. Both reviewers were given image quality assessment forms to assess the image noise, image quality on both axial and coronal images, quality of bowel wall enhancement, and degree of bowel distention at the distal ileum (Supplementary Material 2). Image quality was assessed by comparing the routine CTE of pediatric patients at the authors' institution. Representative images for subjective image quality assessment are shown in Fig. 1.

### Interpretation of bowel findings

Interpretation of the bowel findings was performed by the same radiologists who participated in the subjective image quality assessment. Both reviewers were blinded to the patients' information and clinical and laboratory findings, including the ileocolonoscopic findings. The most severely diseased segment of the terminal ileum was selected by an independent radiologist who participated in the objective image quality assessment, and the chosen images were shown to the reviewers.

Reviewers assessed mural hyperenhancement, wall thickening, mural stratification, ulcers, and perienteric fat stranding. The terminal ileum was defined as the distal 20-cm segment of the ileum from the ileocecal valve. Each finding was subjectively graded on the following five-point scale: 1 = definitely absent, 2 = probably absent, 3 = equivocal, 4 = probably present, and 5 = definitely present. After scoring the imaging findings, the reviewers requested to determine the bowel disease activity following the criteria described in a previous study^22^.

### Reference standard

The results of CTE in determining active small bowel CD were compared with ileocolonoscopic findings as reference standards. Ileocolonoscopy was performed by a board-certified pediatric gastroenterologist with 12 years of pediatric endoscopy experience. Approximately 20 cm of the terminal ileum was evaluated via ileocolonoscopy. The simple endoscopic score was adapted to assess disease severity by analyzing the size of ulcers, the surface area involved by disease and ulceration, and the presence of stenosis^23^. Endoscopic severity was decoded as follows: 0–2, no active disease; 3–6, mild disease activity; and > 7, severe disease activity^23^. Active disease was defined when the endoscopic score was ≥ 3, and it was used as a reference standard to assess diagnostic performance of CTE in determining active small bowel CD. The CTE images were blinded to the gastroenterologist.

### Radiation dose

CTE images were uploaded to an automated dose management system (Radimetrics, Bayer Healthcare) to analyze the radiation dose. CT dose metrics, including volume CT dose index (CTDI_vol_), dose-length product (DLP), size-specific dose estimation (SSDE), and effective dose were automatically retrieved from the dose management software. The SSDE was estimated based on the water-equivalent diameter and conversion factor provided by the American Association of Physicists in Medicine^24^. The effective dose was calculated using a built-in mathematical phantom, age and sex of patients, and tissue-weighting factors derived from the International Commission of Radiological Protection^25^. The effective diameter of the body was retrieved using the dose management system. The effective diameter represents the assumptive circular diameter of the patient.

### Statistical analysis

Patient characteristics were expressed using descriptive statistics. Comparisons of variables were performed by either an independent *t*-test or the Mann–Whitney *U* test for parametric and nonparametric variables, respectively. Comparisons of proportions were performed using the chi-square test. The scores were reclassified into three categories to assess readers' confidence in interpreting bowel findings (i.e., high confidence scores 1 and 5, intermediate confidence scores 2 and 4, and a low confidence score 3)^26^. Readers’ confidence was compared using the Freeman–Halton extension of Fisher's exact test^27^. Bowel findings with scores of 4 and 5 were decoded as positive, while 1, 2, and 3 were decoded as negative for each small bowel finding to assess the overall percentage agreement. A 2 × 2 table was generated to evaluate the diagnostic performance of CTE in determining active small bowel CD. Statistical analysis was performed using MedCalc® Statistical Software version 19.7 (MedCalc Software Ltd., Ostend, Belgium) and SPSS Statistics for Windows version 25.0 (IBM Corp, Armonk, NY, USA). The findings were considered statistically significant when the *P* value was < 0.05.

## Results

### Patient characteristics

Between July 2015 and February 2018, 21 enrolled patients underwent CTE and ileocolonoscopy. All patients underwent CTE within 7 days of ileocolonoscopy. Moreover, no adverse effects were reported on CTE. Three patients allocated to the RD group were excluded from the study due to failure to assess the terminal ileum on endoscopy. Therefore, 18 patients (SD group, *n* = 9; RD group, *n* = 9) were included in this study. The final diagnoses were CD (*n* = 15), intermediate colitis (*n* = 1), irritable bowel syndrome (*n* = 1), or ulcerative colitis (*n* = 1). Patient characteristics are summarized in Table 1. Age, weight, sex, effective diameter, Pediatric CD activity index, C-reactive protein, and fecal calprotectin > 50 mg/kg did not significantly differ between the two groups.

**Table 1:** Summary of patient characteristics.

Variable	All patients (*n* = 18)	Standard-dose group (*n* = 9)	Reduced-dose group (*n* = 9)	*P* value
Age (years)	14.6 ± 2.2 (11–18)	15.2 ± 1.9 (13–18)	14.0 ± 2.4 (11–18)	0.25
Weight (kg)	48.8 ± 10.8 (30–75)	51.8 ± 9.4 (44–75)	45.8 ± 11.7 (30–61)	0.67
Male versus female ratio	11:7	5:4	6:3	0.61
Effective diameter (cm)	21.9 ± 1.9 (19.3–26.5)	22.7 ± 1.7 (20.2–26.5)	21.3 ± 1.9 (19.3–24.3)	0.24
PCDAI^a^	30.3 ± 23.9	31.5 ± 29.9	28.6 ± 23.9	0.85
CRP (mg/L)^b^	2.6 ± 2.6	2.6 ± 2.7	2.6 ± 2.6	0.97
Fecal calprotectin > 50 mg/kg^c^	15	8	7	0.57
**Simple endoscopic score**				
0–2	3	3	0	
3–6	12	5	7	
7–15	3	1	2	

*PCDAI* Pediatric Crohn's disease activity index; *CRP* C-reactive protein.

^a^PCDAI was measured in 15 patients.

^b^CRP level was measured in 18 patients.

^c^Fecal calprotectin levels were measured in 15 patients.

Numbers in parentheses represent a range.

### Image quality analysis

**Figure 2:**
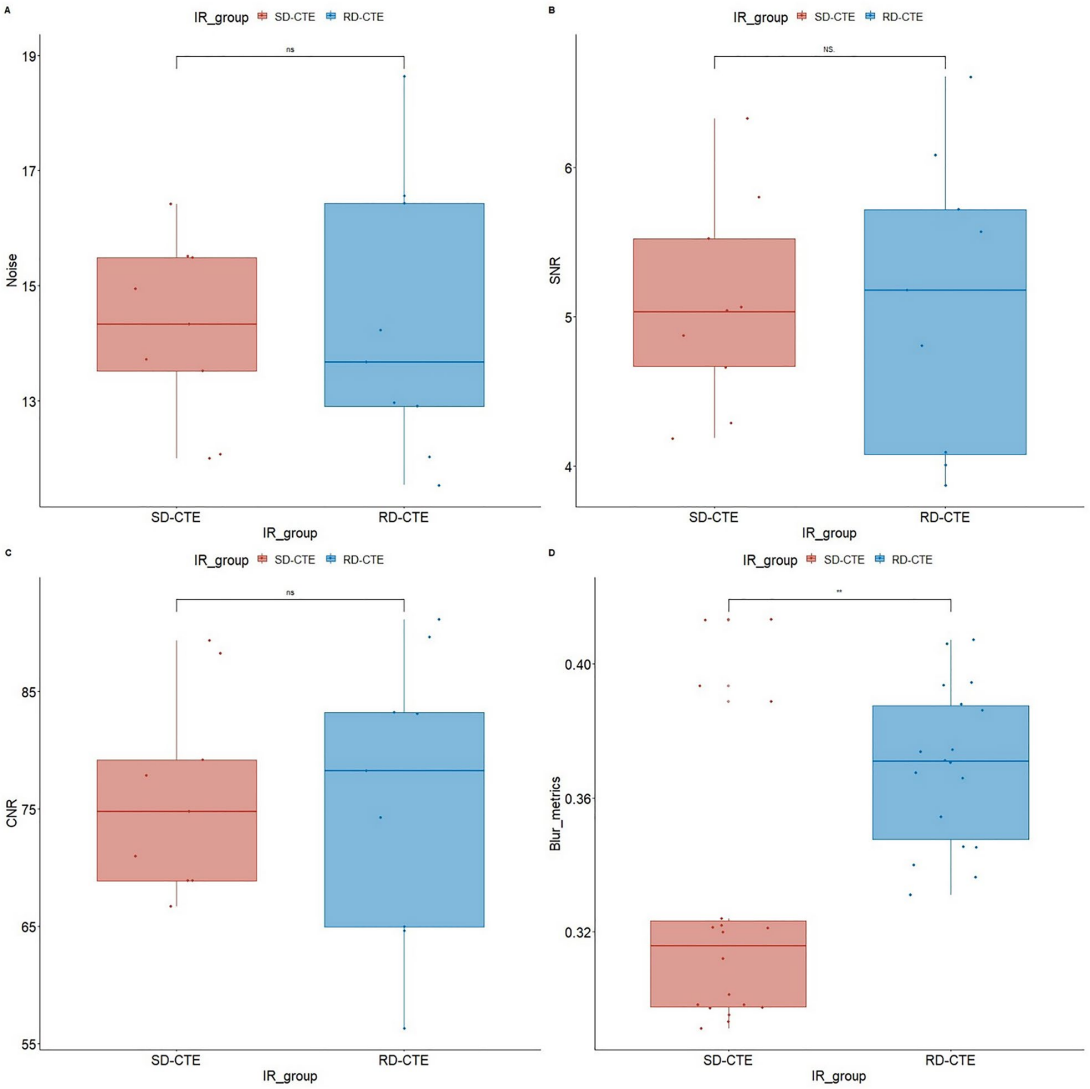
Comparison of the objective image quality score. *P < 0.05, statistically significant; *ns* not statistically significant; *SNR* signal-to-noise ratio; *CNR* contrast-to-noise ratio; *SD-CTE* standard-dose CT enterography; *RD-CTE* reduce-dose CE enterography.

Image noise, SNR, and CNR of the SD-CTE and RD-CTE did not show significant differences (Fig. 2a–c). However, the blur metric value of the RD-CTE was significantly higher than that of SD-CTE (Fig. 2d), suggesting more blurred images in RD-CTE.

**Figure 3:**
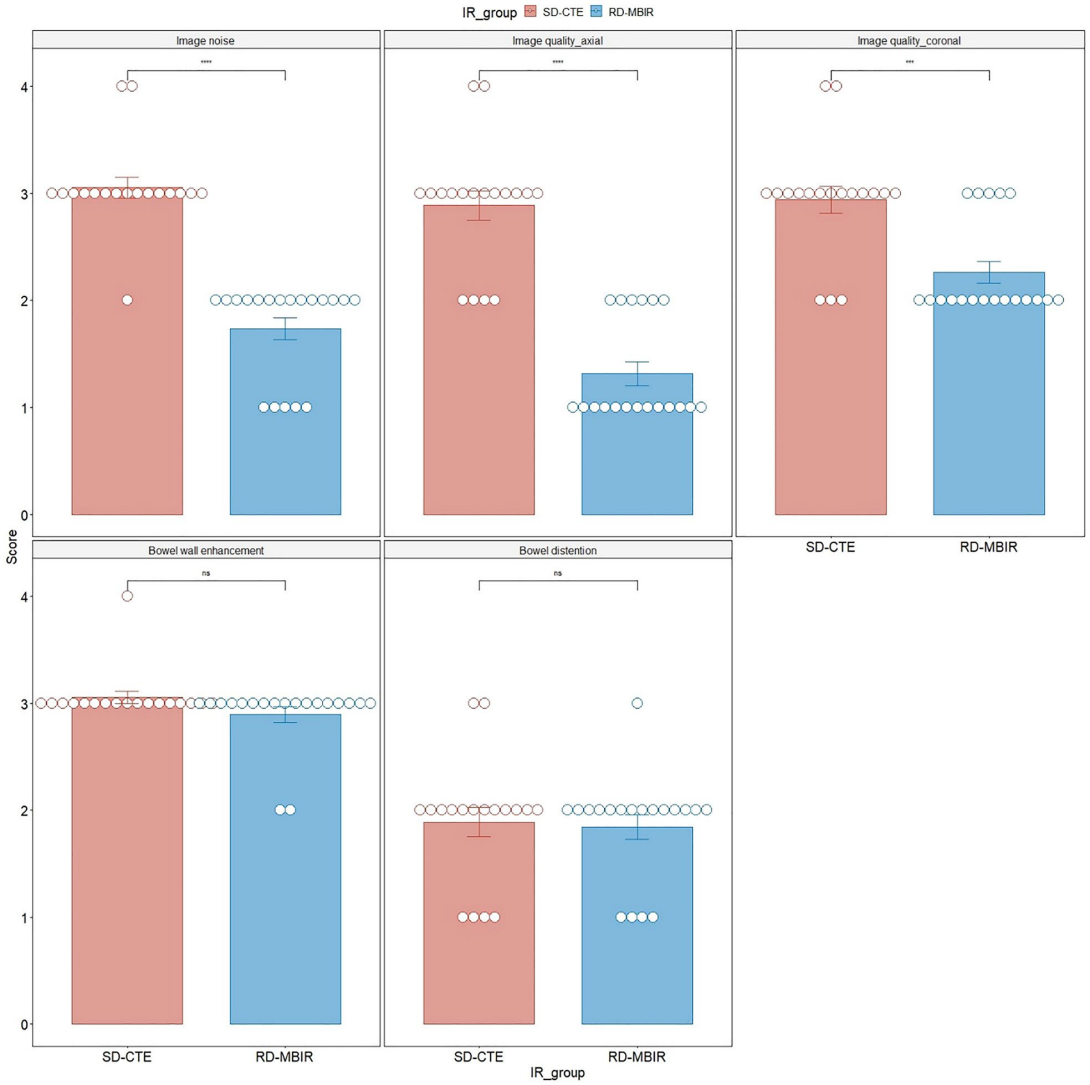
Comparison of the subjective image quality score. asterisks, statistically significant (*P* < 0.05); ns, not statistically significant; SD-CTE, standard-dose CT enterography; RD-CTE; reduce-dose CE enterography.

**Figure 4:**
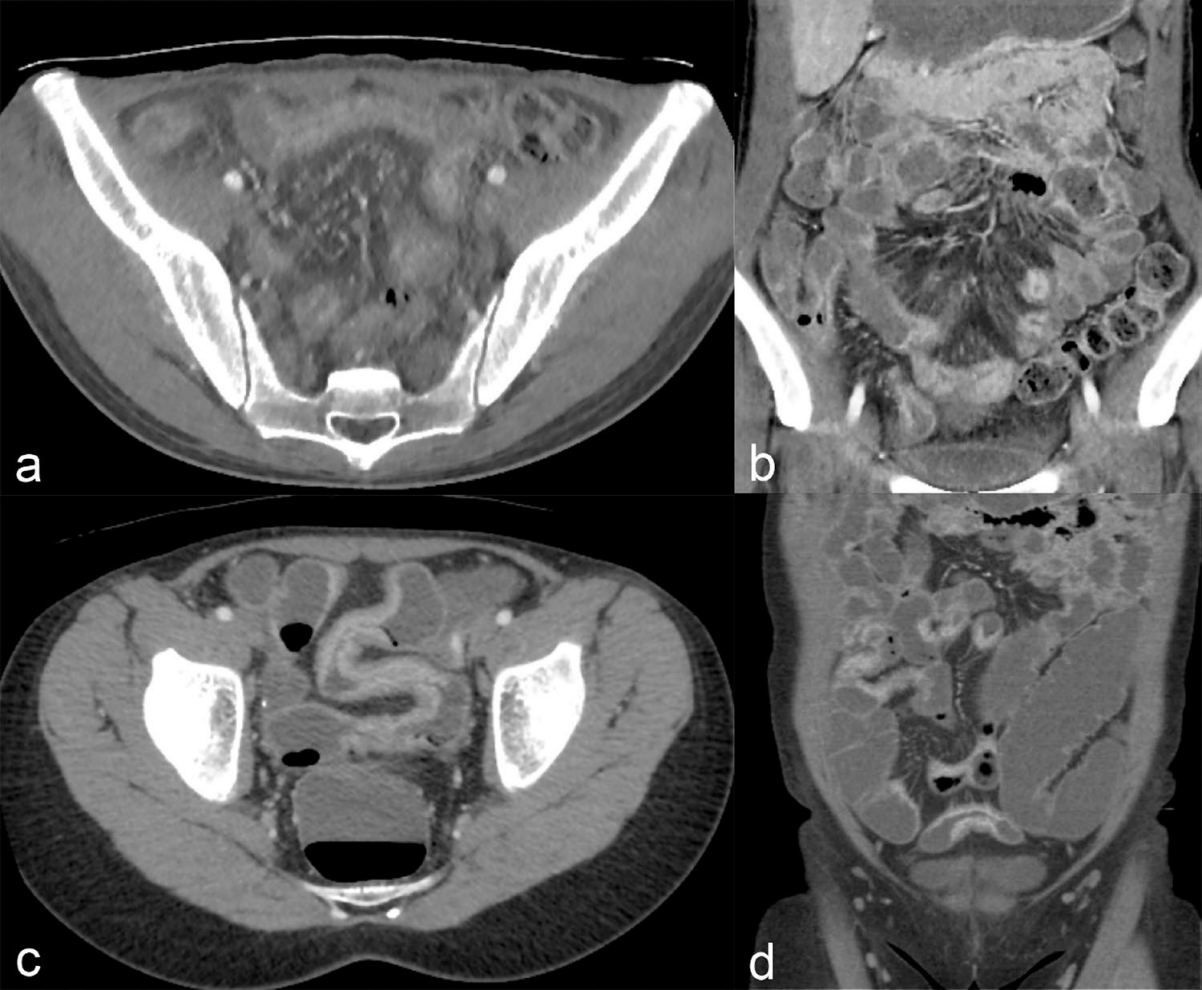
Representative images of (**a**) and (**b**) standard-dose CT enterography and (**c** and **d**) reduced-dose CT enterography in two patients. Both cases show active small bowel Crohn's disease, manifested with mural hyperenhancement, bowel wall thickening, and multiple skin lesions. In the subjective image quality assessment, (**a**, **b**, **c**), and (**d**) represent scores of 2/1, 3/3, 4/3, and 4/3 (reviewers 1/2), respectively.

The subjective image quality scores are shown in Fig. 3. The RD-CTE showed less image noise than SD-CTE; however, the image quality scores of both axial and coronal images were inferior to SD-CTE. This result was due to blotchy image texture in the RD-CTE. When comparing image quality between the axial and coronal images of the RD-CTE, coronal images scored higher than axial images (*P* < 0.01), whereas no intergroup difference was noted in SD-CTE (*P* = 0.75; Fig. 4). In addition, the degree of bowel wall enhancement and bowel distention did not show intergroup differences.

### Interpretation of bowel findings and analysis of the diagnostic performance

Table 2 summarizes the readers' confidence and overall percentage agreement in interpreting bowel findings. No intergroup difference was noted between SD-CTE and RD-CTE in interpreting mural hyperenhancement and wall thickening. However, reader confidence was inferior in the RD-CTE in interpreting mural stratification, ulcer, and perienteric fat stranding.

**Table 2:** Reader confidence and overall percentage agreement in interpreting bowel findings between SD-CTE and RD-CTE.

		SD-CTE (*n* = 18)	RD-CTE (*n* = 18)	*P* value
Mural hyperenhancement	High	14	18	0.05
	Intermediate	4	0	
	Low	0	0	
	Overall percent agreement	9/9	9/9	
Wall thickening	High	15	18	0.11
	Intermediate	3	0	
	Low	0	0	
	Overall percent agreement	9/9	9/9	
Mural stratification	High	8	9	0.03
	Intermediate	10	4	
	Low	0	5	
	Overall percent agreement	9/9	8/9	
Ulcer	High	14	2	< 0.01
	Intermediate	4	9	
	Low	0	7	
	Overall percent agreement	7/9	6/9	
Perienteric fat stranding	High	16	5	< 0.01
	Intermediate	2	10	
	Low	0	3	
	Overall percent agreement	9/9	7/9	

*SD-CTE* standard-dose CT enterography; *RD-CTE* reduced-dose CT enterography.

Total number of each group (*n* = 18) is due to double assessment by two readers.

The diagnostic performance of detecting active disease is summarized in Table 3. Both SD-CTE and RD-CTE correctly diagnosed active disease, except for one case of mild endoscopic severity in the SD-CTE group.

**Table 3:** Cross-table of the SD-CTE and RD-CTE with endoscopic reference.

		Endoscopic severity		
		No active disease	Mild	Severe
SD-CTE	Active disease ( +)	0	4	1
	Active disease ( −)	3	1	0
RD-CTE	Active disease ( +)	0	6	3
	Active disease ( −)	0	0	0

*SD-CTE* standard-dose CT enterography; *RD-CTE* reduced-dose CT enterography.

### Radiation dose

**Figure 5:**
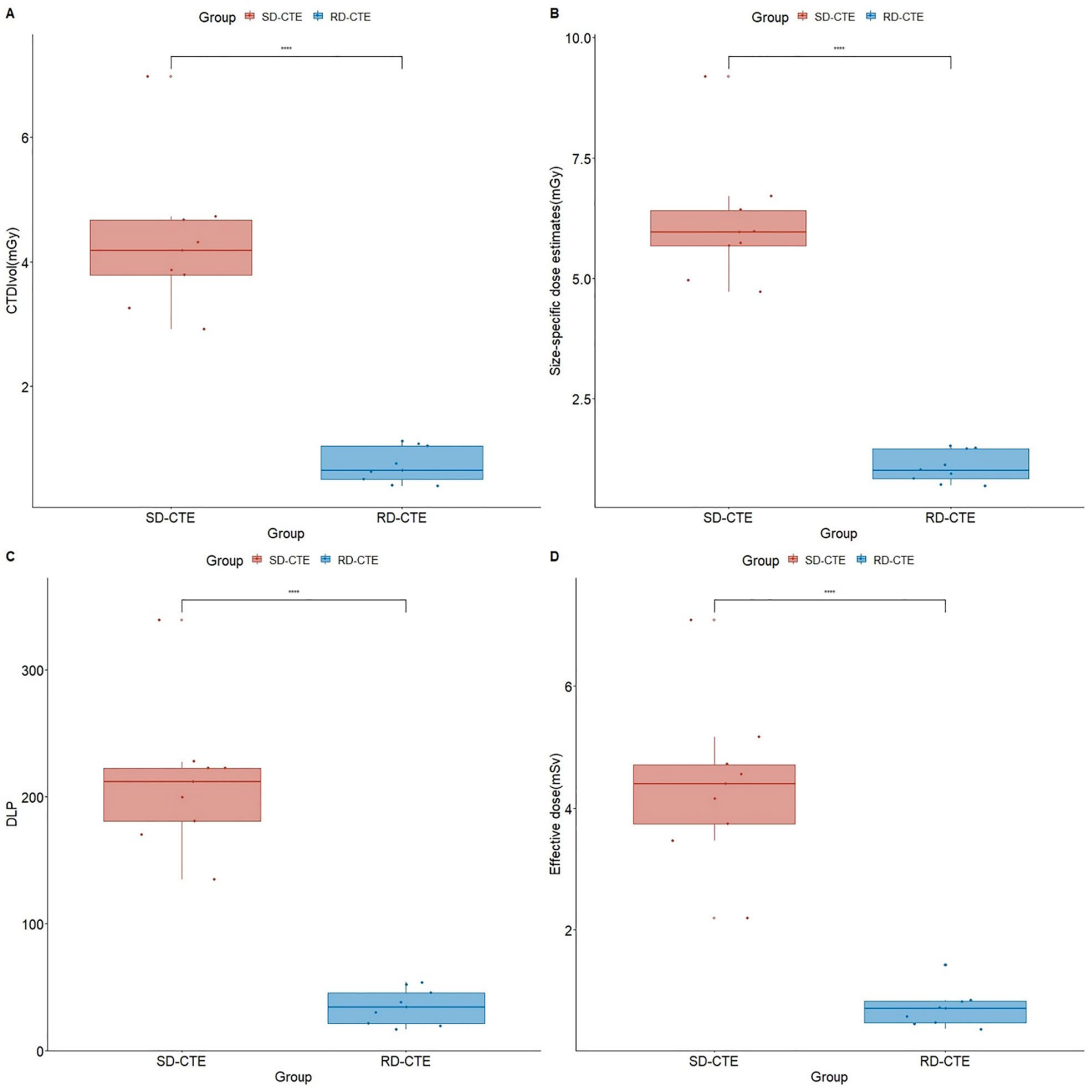
Comparison of the radiation dose. *P < 0.05 statistically significant; *CTDI*_*vol*_ volume CT dose index; *DLP* dose-length product; *SD-CTE* standard-dose CT enterography; *RD-CTE* reduce-dose CE enterography.

Figure 5 reveals the comparisons of radiation dose between two groups. The mean values of radiation dose metrics of the standard-versus RD group were 4.3 versus 0.74 mGy, 6.1 versus 1.1 mGy, 211.9 versus 34.5 mGy∙cm, and 4.4 versus 0.7 mSv for CTDI_vol_, SSDE, DLP, and effective dose, respectively. The dose reduction rates of the RD group were 82.9%, 82.3%, 83.7%, and 83.7% for CTDI_vol_, SSDE, DLP, and effective dose, respectively.

## Discussion

Overall, the RD-CTE using 80 kVp combined with MBIR achieved > 80% dose reduction compared with SD-CTE while maintaining comparable diagnostic accuracy in detecting active small bowel CD in the terminal ileum. The average effective dose of the RD-CTE was 0.7 mSv. Although the MBIR technique drastically reduced quantum mottles in the RD-CTE, it affected image quality due to blotchy image texture and readers' confidence, especially in detecting mural stratification, ulcers, and perienteric fat stranding.

A false-negative case in the SD-CTE group was noted; this misdiagnosis may have resulted from the character of the disease rather than the image quality. The endoscopic severity was mild along with several shallow ulcers in the terminal ileum; however, the depth of the ulcers was too shallow to be detected on the CTE. In addition, this case did not show mural hyperenhancement and bowel wall thickening on CTE, unlike the other 10 of 11 cases with mild endoscopic severity. Interestingly, the determination of the disease activity depended mainly on the presence of the mural hyperenhancement and bowel wall thickening in the current study; these two findings are always present in cases with mild and severe endoscopic severity except for the aforementioned false-negative case.

Radiation exposure of pediatric patients has always been a concern because children are at great risk for radiation-induced cancer from ionizing radiation due to organ susceptibility and long life expectancy^28^. This potential hazard of ionizing radiation is particularly important in pediatric patients with inflammatory bowel disease because this patient group is more likely to undergo multiple CT scans over the lifetime. Desmond et al.^29^ reported that CT accounted for 77.2% of radiation exposure in patients with inflammatory bowel disease, and the cumulative dose of 75 mSv was exceeded in 15.5% of those patients. Young age was a significant factor for a high cumulative dose with a hazard ratio of 2.1. In a more recently published meta-analysis, the pooled estimated proportion of high radiation doses was 8.4%, and patients with CD were more likely to be exposed to high cumulative doses than patients with ulcerative colitis^30,31^.

Regarding the radiation exposure from the CTE, MRE may be the best imaging modality for pediatric CD patients in many child care centers. However, CTE is still preferred in specific clinical scenarios because of its higher spatial resolution, less motion artifact, shorter scan time, lower cost, and higher scanner availability than MRE. According to the American College of Radiology appropriateness criteria, both CTE and MRE are rendered as usually appropriate for initial imaging for children with suspected CD, suspected acute exacerbation of known CD, and disease surveillance or monitoring therapy^32^. Appropriately optimized CTE protocol with the application of modern dose reduction techniques (e.g., automatic exposure control, iterative reconstruction, lowering tube voltage, and high-pitch acquisition in conjunction with appropriate justification) can significantly reduce the overall radiation dose^14,17,33,34^.

As previously noted, the downside of the iterative reconstruction is an unfamiliar image texture manifested as *pixelated* or *blotchy* images, particularly in the MBIR technique^12–16^. The subjective image quality score of the RD-CTE group was inferior to the SD-CTE because of the excessively pixelated image texture in the former. In addition, in the objective assessment of image sharpness with blur metric analysis, the RD-CTE showed inferior image sharpness to the SD-CTE with adaptive statistical iterative reconstruction with a blending factor of either 50%. This inherent MBIR characteristic can also affect the diagnostic accuracy of small-and low-contrast lesions. Although previous studies revealed MBIR usefulness in detecting focal hepatic lesions or renal calculi^9,35^, recently published studies pointed to compromised diagnostic performance and reader confidence of RD CT scan combined with MBIR technique for detection of low-contrast liver lesions in adult patients^36,37^.

Similarly, the results of the current study also showed reduced readers’ diagnostic confidence in interpreting mural stratification, ulcer, and perienteric fat stranding in the RD-CTE group while retaining comparable confidence to SD-CTE in detecting wall thickening and mural hyperenhancement. Excessively pixelated image texture and decreased image sharpness were believed to be responsible for reduced reader confidence in detecting small, low-contrast, and ill-defined lesions. Interestingly, the subjective image quality score of the coronal image was better than that of the axial images, although the exact reason behind this observation was not determined in this study. Moreover, the blotchy and blurred image texture was slightly alleviated in the coronal reconstructed images compared with the axial images in MBIR (Fig. 4). This image character was beneficial for interpreting CTE because coronal images are preferred for interpreting enterography images because they ensure a better anatomical perception of bowel segments.

Deep learning-based reconstruction (DLR) techniques have recently become clinically available. DLR can provide substantial noise suppression while providing fast reconstruction speed, natural and fine image texture even at the lower dose settings, and high spatial resolution^33^. It also has a dose reduction capability for both high- and low-contrast object tasks, whereas the low-contrast object detectability can be challenging in iterative recontructions^33,37,38^. Regarding the low-contrast character of bowel disease, the small size of the lesion, and the complex course of the bowel structures, DLR is believed to have an excellent potential to be the best reconstruction method for CTE, achieving low-dose and good image quality.

The current study did not show superior tissue contrast and bowel wall enhancement in the RD-CTE even though the reduced-dose protocol used was 80 kVp. Moreover, both CNR and subjective scores for bowel enhancement were not significantly different between the two groups. The difference in CTE contrast may be mitigated in the current study because 100 kVp is already applied in the SD protocol. In the literature, however, the use of low kVp in CT scanning helps increase the contrast of the iodine contrast media^4–6,34,39^, and mucosal hyperenhancement and mural stratification of inflamed bowel can be more pronounced with radiation dose reduction^4,6^. Therefore, the application of low kVp in CTE should be tailored to the patient size and availability of denoizing algorithms (e.g., iterative reconstruction).

This study has several limitations. First, early study termination due to difficulty in patient enrolment resulted in a small number of included patients and asymmetric distribution of active disease in both groups. Therefore, the statistical significance of diagnostic performance and sufficient statistical power could not be achieved. Second, different characters of patients between two groups or within groups may have resulted in bias in assessing subjective image analysis, reader confidence, and diagnostic performance. Although no statistical differences were noted between the two groups regarding body weight, the wide range of bodyweight distribution may have affected image quality and performance of automatic exposure control. However, paired lesion-by-lesion comparison of SD-CTE and RD-CTE in the same patient was not performed due to ethical concerns related to the potential hazard from radiation exposure in pediatric patients. Third, the ileocolonoscopy was used for the reference standard in determining the presence of active disease; however, it is somewhat equivocal how the endoscopic findings were correlated with CTE findings, especially for the perienteric fat stranding. Evaluation of extraenteric manifestations was beyond the scope of the study, and the occurrence of substantial abnormalities in abdominal solid organs is unlikely in pediatric patients. Last, disease activity was assessed only in the terminal ileum. Even though the large bowel disease can be evaluated in CTE, endoscopy plays the primary role in determining disease activity of the large bowel. In addition, inconsistent and suboptimal distention of the large bowel in the pediatric population is often experienced.

In conclusion, the current study showed that RD-CTE using 80 kVp combined with MBIR technique has comparable diagnostic accuracy to that of the SD-CTE in determining active disease of the terminal ileum in pediatric CD patients. Although the RD-CTE with MBIR showed excellent denoizing performance with approximately 80% dose reduction, image quality and reader confidence in detecting mural stratification, ulcer, and perienteric fat stranding were compromised. Further studies are needed to evaluate these findings on larger cohorts of patients using various dose reduction rates and artificial intelligence technologies.

## Supplementary Information

Supplementary Information.
