# Supplementary material for: Image quality and diagnostic accuracy of reduced-dose computed tomography enterography with model-based iterative reconstruction in pediatric Crohn’s disease patients
Source: Sci Rep. 2022 Feb 9;12:2147. doi: 10.1038/s41598-022-06246-z (PMC8828853; doi:10.1038/s41598-022-06246-z)
Supplement: Supplementary file 1 — Supplementary Information. [file 41598_2022_6246_MOESM1_ESM.docx]

**Supplementary materials**

**Supplementary material 1. The setting of peak kilovoltage and noise index of computed tomography enterography.**

| Protocol | Weight | 30-39.9 kg | 40-49.9 kg | 50-59.9 kg | >60 kg |
| --- | --- | --- | --- | --- | --- |
| Group A | kVp | 100 | 100 | 100 | 120 |
|  | Noise index | 15 | 15 | 16 | 16 |
|  | mA Range | 20-400 | 20-400 | 20-400 | 20-400 |
| Group B | kVp | 80 | 80 | 80 | 80 |
|  | Noise index | 35 | 35 | 36 | 36 |
|  | mA Range | 20-400 | 20-400 | 20-400 | 20-400 |

kVp, peak kilovoltage

**Supplementary material 2. Reader assessment form for subjective image quality analysis.**

| Image quality (axial) | | |
| --- | --- | --- |
| 1 |  | Inadequate for diagnosis |
| 2 |  | Worse than routine examination, but interpretable |
| 3 |  | Similar to routine examination |
| 4 |  | Better than routine examination |
| Image quality (coronal) | | |
| 1 |  | Inadequate for diagnosis |
| 2 |  | Worse than routine examination, but interpretable |
| 3 |  | Similar to routine examination |
| 4 |  | Better than routine examination |
| Please provide the reason for image quality 1 or 2 | | |
|  | | |
| Bowel wall enhancement | | |
| 1 |  | Inadequate for diagnosis |
| 2 |  | Worse than routine examination, but interpretable |
| 3 |  | Similar to routine examination |
| 4 |  | Better than routine examination |
| Image noise | | |
| 1 |  | Too little noise |
| 2. |  | Less than the usual noise |
| 3 |  | Usual noise |
| 4 |  | Excessive noise not affecting diagnostic interpretation |
| 5 |  | Excessive noise inadequate for diagnostic interpretation |
| Degree of bowel distention at the distal ileum | | |
| 0 |  | No distention |
| 1 |  | Poor distention |
| 2 |  | Good distention |
| 3 |  | Optimal distention |
